# Changing glucocorticoid action: 11β-Hydroxysteroid dehydrogenase type 1 in acute and chronic inflammation

**DOI:** 10.1016/j.jsbmb.2013.02.002

**Published:** 2013-09

**Authors:** Karen E. Chapman, Agnes E. Coutinho, Zhenguang Zhang, Tiina Kipari, John S. Savill, Jonathan R. Seckl

**Affiliations:** a University/BHF Centre for Cardiovascular Sciences, The Queen's Medical Research Institute, University of Edinburgh, 47 Little France Crescent, Edinburgh EH16 4TJ, UK; b MRC Centre for Inflammation Research, The Queen's Medical Research Institute, University of Edinburgh, 47 Little France Crescent, Edinburgh EH16 4TJ, UK

**Keywords:** 11β-HSD, 11β-hydroxysteroid dehydrogenase, H6PD, hexose-6-phosphate dehydrogenase, TNF-α, tumour necrosis factor-α, LPS, lipopolysaccharide, IL, interleukin, C/EBP, CCAAT/enhancer binding protein, NF-κB, nuclear factor kappa-light-chain-enhancer of activated B cells, EGR-1, early growth response-1, HPA, hypothalamic–pituitary–adrenal, MCP, monocyte chemotactic protein, VCAM, vascular cell adhesion molecule, Glucocorticoid, Mineralocorticoid, 11β-Hydroxysteroid dehydrogenase, Macrophage, Inflammation, Arthritis

## Abstract

Since the discovery of cortisone in the 1940s and its early success in treatment of rheumatoid arthritis, glucocorticoids have remained the mainstay of anti-inflammatory therapies. However, cortisone itself is intrinsically inert. To be effective, it requires conversion to cortisol, the active glucocorticoid, by the enzyme 11β-hydroxysteroid dehydrogenase type 1 (11β-HSD1). Despite the identification of 11β-HSD in liver in 1953 (which we now know to be 11β-HSD1), its physiological role has been little explored until recently. Over the past decade, however, it has become apparent that 11β-HSD1 plays an important role in shaping endogenous glucocorticoid action. Acute inflammation is more severe with 11β-HSD1-deficiency or inhibition, yet in some inflammatory settings such as obesity or diabetes, 11β-HSD1-deficiency/inhibition is beneficial, reducing inflammation. Current evidence suggests both beneficial and detrimental effects may result from 11β-HSD1 inhibition in chronic inflammatory disease. Here we review recent evidence pertaining to the role of 11β-HSD1 in inflammation.

This article is part of a Special Issue entitled ‘CSR 2013’.

## Highlights

► 11β-HSD1 converts inert glucocorticoids into active forms, amplifying glucocorticoid action. ► 11β-HSD1 is markedly induced by pro-inflammatory cytokines. ► 11β-HSD1 deficiency/inhibition worsens acute inflammation. ► 11β-HSD1 inhibition reduces inflammation in obesity or atherosclerosis. ► An increased angiogenic response may underlie some of the benefits.

## 1 Introduction

The discovery of the anti-inflammatory effects of cortisone, a glucocorticoid hormone, by Hench and colleagues in the 1940s, opened the door to the longest and most successful drug development programme in history. Glucocorticoids remain the most widely prescribed treatment for inflammatory disease. They potently affect both immune and non-immune cells, shaping their responses. Glucocorticoid actions are highly dependent on context and can be very different during acute and chronic inflammation. In the short term at least, many of their effects promote the resolution of inflammation. Several years ago, we hypothesised that the glucocorticoid metabolising enzyme, 11β-hydroxysteroid dehydrogenase type 1 (11β-HSD1), is induced early during an inflammatory response and shapes its subsequent trajectory [1]. How well has that hypothesis stood the test of time? Reasonably well as it turns out, but not in quite the way we had envisaged.

### 1.1 Glucocorticoids and inflammation

Synthetic glucocorticoids exert potent anti-inflammatory and immunosuppressive effects and are widely prescribed to treat both acute and chronic inflammation. Yet the well known side effects of glucocorticoid excess include type 2 diabetes, visceral obesity, hypertension and atherosclerosis which are themselves, somewhat paradoxically, inflammatory conditions. Quite how glucocorticoids provoke inflammatory metabolic diseases at the same time as suppressing chronic inflammatory conditions such as rheumatoid arthritis or inflammatory bowel disease remains unclear. It is likely to involve more complex mechanisms than the commonly held view that the “adverse” metabolic effects involve gene activation by glucocorticoid receptor (GR), whereas the “beneficial” anti-inflammatory effects rely on gene repression. Fully understanding how glucocorticoids cause “metabolic inflammation” will be crucial for the development and optimal exploitation of future anti-inflammatory therapies, which could manipulate glucocorticoid action in a more sophisticated manner than current therapies.

Understanding the role of endogenous glucocorticoids during inflammation is key to achieving this aim. Endogenous glucocorticoids are vital to survive trauma or certain bacterial infections; they suppress pro-inflammatory cytokine production, binding to GR in immune cells to prevent potentially lethal overshoot of immune responses [2], [3]. Acutely, circulating pro-inflammatory cytokines are a potent stimulus to the hypothalamic–pituitary–adrenal (HPA) axis to increase endogenous glucocorticoid production [4], [5]. However, this normal response is lost or attenuated in chronic inflammation [6]. In this respect, the treatment of chronic inflammatory disease with exogenous glucocorticoids can be regarded as replacement therapy for an inadequate endogenous glucocorticoid response [7].

Acute inflammation is an immediate response of the body to injury or infection that serves to remove the injurious stimulus, then restore homeostasis by removal of dead and damaged cells/tissues and engagement of repair processes. It is initiated at the site of injury by the release of proinflammatory mediators such as bioactive amines, lipids and cytokines: typically tumour necrosis factor (TNF)-α and interleukin (IL)-1. These cause vasodilation, increase vascular permeability allowing exudation of plasma, and elicit leucocyte recruitment, activation and emigration from the microcirculation to the damaged tissue. The initial response is typically predominated by neutrophils, which are replaced by monocytes/macrophages during the resolution and repair stages. Resolution of acute inflammation requires the engagement of mechanisms early in the inflammatory response that shape the subsequent resolution (reviewed in Refs. [8], [9], [10]). Chronic inflammation results from persistence of the initiating stimulus with associated lymphocyte and macrophage activation. Excessive tissue damage contributes to continuing inflammation, failure of resolution and dysregulated repair processes such as angiogenesis and fibrosis and can thus form a “vicious” cycle. Whilst acute inflammation frequently occurs and is contained entirely at the local level, chronic inflammation invariably involves a systemic response.

Glucocorticoids limit acute inflammation. They repress a large number of proinflammatory genes, including pro-inflammatory cytokines and chemokines, cell adhesion molecules and enzymes involved in the initiation and/or maintenance of inflammation, many of which are over-expressed in chronic non-resolving inflammation. Conversely, they activate a number of genes encoding anti-inflammatory mediators, such as IL-10 and annexin I (reviewed in Refs. [11], [12], [13]). Thus, acutely, glucocorticoids inhibit the initial vasodilation and increased vascular permeability during inflammation. They also alter the balance between survival and apoptosis of leukocytes as well as their distribution between the circulation and immune tissues and they decrease leucocyte emigration into sites of injury [13], [14], [15], [16], [17], [18]. Importantly, glucocorticoids potently influence the differentiation and phenotype of immune cells, especially monocytes/macrophages and T lymphocytes, thereby polarising, or shaping, immune responses [19]. Glucocorticoid treatment of human monocytes promotes an anti-inflammatory, pro-resolution phenotype, characterised by high migratory and phagocytic capacity, expression of CD163 (haemoglobin scavenger receptor) and high production of IL-10 [20], [21], [22], [23]. Similarly, in mice, pro-resolving macrophage functions are enhanced by glucocorticoid treatment [24], [25], thus shaping the trajectory of an inflammatory response and its outcome. Because glucocorticoids inhibit production of “Th1” cytokines, which promote a cell-mediated immune response (activation of phagocytes, antigen-specific T lymphocytes) whilst preserving or promoting “Th2” cytokine production (aiding antibody production), they also shape the adaptive immune response.

Most research on the anti-inflammatory actions of glucocorticoids has utilised dexamethasone, a potent synthetic glucocorticoid with powerful immunosuppressive properties. However, the endogenous glucocorticoids, cortisol (the main glucocorticoid in humans) and corticosterone (in rats and mice), are immunomodulatory rather than immunosuppressive [14], [26], particularly when administered at physiologically relevant concentrations. Indeed, low doses of corticosterone stimulate whereas higher doses suppress macrophage activity [27]. This could, in part, reflect the higher affinity binding of endogenous glucocorticoids to the mineralocorticoid receptor (MR) (dexamethasone poorly activates MR [28]) than to GR as both are expressed in macrophages [27], [29], [30]. However, whereas knock-down or antagonism of GR in macrophages abrogates responses to both high and low doses of corticosterone, knock-down or antagonism of MR has little effect [27], suggesting GR-mediated effects, at least in the rat macrophages tested. The interplay between GR and MR in macrophage function and polarisation is likely to be complex (see below).

### 1.2 11β-Hydroxysteroid dehydrogenases modulate glucocorticoid action

Endogenous glucocorticoids differ from dexamethasone in another important respect; dexamethasone is not inactivated by 11β-HSD activity [31] whereas endogenous glucocorticoids are substrates for the 11β-HSDs, which are important modulators of physiological glucocorticoid action [32]. The 11β-HSD “shuttle” interconverts active glucocorticoids (cortisol, corticosterone) with their 11-keto forms (cortisone, 11-dehydrocorticosterone), which bind poorly to receptors and are therefore intrinsically inert. In intact cells, 11β-HSD1 exhibits oxo-reductase activity, converting cortisone and 11-dehydrocorticosterone into active cortisol and corticosterone respectively, increasing intracellular glucocorticoid levels. In contrast, 11β-HSD2 is exclusively a dehydrogenase, inactivating cortisol and corticosterone. Expression of 11β-HSD2 is largely restricted to mineralocorticoid-target tissues, most notably the distal nephron of the kidney where it protects the non-selective MR from activation by glucocorticoids, conferring aldosterone-specificity upon MR, which is otherwise a high affinity glucocorticoid receptor [33], [34]. Of the synthetic glucocorticoids in widespread use as anti-inflammatory drugs, it is worth noting that some, including prednisone/prednisolone, are excellent substrates for the 11β-HSDs.

**Fig. 1:**
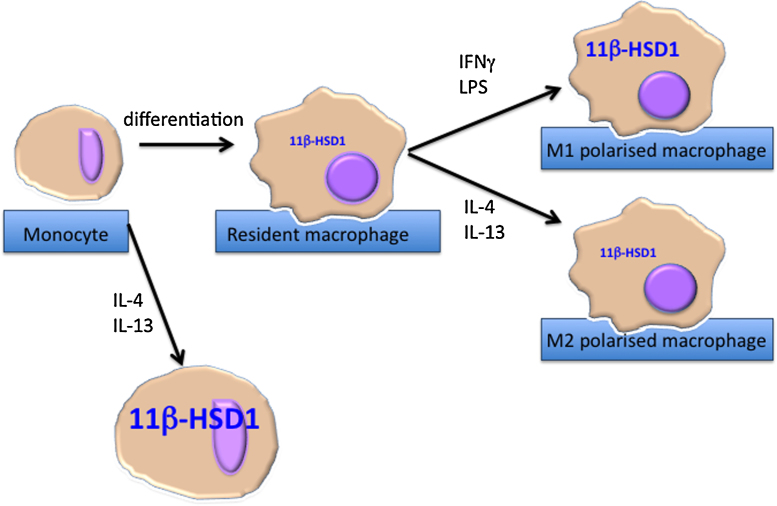
11β-HSD1 is induced upon macrophage differentiation. Expression of 11β-HSD1 is negligible in human monocytes, but is induced on differentiation into macrophages. Polarisation of macrophages to an M1 phenotype further induces 11β-HSD1 whereas polarisation to an M2 phenotype has no further effect on expression. Differentiation of monocytes into macrophages in the presence of IL-4 and/or IL-13 further induces 11β-HSD1 (see text for details).

11β-HSD1 is widely expressed, including in immune cells, where its activity is dynamically regulated depending on cell activation state (reviewed in Ref. [35]). 11β-HSD1 is up-regulated upon activation of monocytes/macrophages, neutrophils or lymphocytes [35], [36] (and see Fig. 1). Circulating leukocytes in mice and healthy humans do not express 11β-HSD2 [1], [37]. Both 11β-HSD isozymes are regulated by pro-inflammatory signalling in non-immune cells (see below for details).

### 1.3 11β-HSD1 expression in monocytes/macrophages depends on cell activation state

Monocytes and macrophages are essential during an inflammatory response. In response to diverse environmental signals, “resting” or naïve macrophages adopt distinct phenotypes. These are broadly categorised based on *in vitro* experiments into two states, M1 (or classically activated) and M2 (or alternatively activated) (reviewed in Refs. [38], [39]). M1 macrophages, induced by interferon-γ and Toll-like receptor (TLR) activation (*e.g.* by lipopolysaccharide, LPS), are vital for host defence, expressing pro-inflammatory cytokines, inducible nitric oxide synthase (iNOS) and demonstrating strong microbicidal activity. M2 macrophages, polarised with IL-4 and/or IL-13, restore homeostasis in the repair phase of inflammation. They are also vital for parasite elimination. Other stimuli induce M2-like anti-inflammatory phenotypes, distinct from IL-4/IL-13 polarised macrophages. Macrophage phenotype *in vivo* may be more complex and heterogeneous [40], especially macrophages with M2-like characteristics, reflecting the diversity of signalling and context *in vivo*. Glucocorticoids restrain M1 macrophages, dampening pro-inflammatory cytokine expression, and in naïve monocytes/macrophages, induce a highly phagocytic, highly motile, M2-like phenotype [21], [24], [41]. Conditional deletion of GR in macrophages increases pro-inflammatory cytokine production and mortality following LPS administration [3], [42]. Conversely, conditional deletion of MR in macrophages promotes polarisation to an alternatively activated (M2) phenotype [43], suggesting a possible reciprocal relationship between GR and MR activation in macrophages. There is therefore considerable potential for 11β-HSD1 (which can potentially supply ligand to either receptor) to modulate monocyte/macrophage phenotype by increasing intracellular glucocorticoid levels, even in the absence of elevated circulating glucocorticoid levels.

**Fig. 2:**
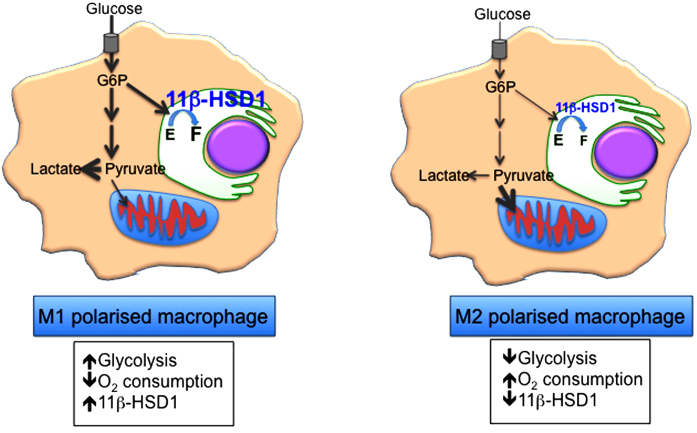
Macrophage polarisation is associated with a switch in energy metabolism. M1 macrophages show a predominantly glycolytic metabolism. High levels of glucose-6-phosphate (G6P) may ensure a ready supply of NADPH cofactor to 11β-HSD1, driving high conversion of cortisone (E) to cortisol (F). M2 polarised macrophages are oxidative, with lower levels of glycolysis and lower levels of 11β-HSD1 converting E to F. Whether changes in energy metabolism drive changes in macrophage 11β-HSD1 expression is currently unknown (see text for details).

Expression of 11β-HSD1 is low in circulating mouse leukocytes but is higher in macrophages [44]. Though negligible in non-stimulated human monocytes, 11β-HSD1 expression is induced upon differentiation into resting or naïve (*i.e.* unstimulated) macrophages [37]. M1 polarisation of naive macrophages with LPS further induces 11β-HSD1 (Figure 1). In contrast, polarisation to an M2 phenotype with IL-4 has little effect on 11β-HSD1 expression [45], [46]. However, in human monocytes differentiated into macrophages in the presence of IL-4 (which may induce a distinct anti-inflammatory macrophage phenotype from M2 polarisation of resting macrophages), 11β-HSD1 activity is as high or higher than in M1, and is further increased by peroxisome proliferator-activated receptor (PPAR)-γ activation [47]. In contrast, in mouse bone marrow-derived macrophages (resting macrophages) PPARγ agonists down-regulate 11β-HSD1 expression [47]; whether this reflects a mouse/human species difference or the different macrophage phenotypes (resting mouse macrophages *versus* human macrophages differentiated in the presence of IL-4) is currently unclear. Nevertheless these studies illustrate a complex dependence of 11β-HSD1 expression upon macrophage activation state. The significance is currently unknown but might reflect (or influence) differences in energy metabolism between glycolytic M1 and oxidative M2 macrophages [48], [49]. Recent evidence suggests manipulation of glucose metabolism in macrophages directly alters polarisation [49]. Whether alterations in 11β-HSD1 expression influence macrophage glucose metabolism, for example through the coupling of 11β-HSD1 oxo-reductase activity to hexose 6-phosphate activity in the endoplasmic reticulum (see below) is an important question to address as it may directly affect polarisation or the extent of activation of macrophages. Dynamic regulation of 11β-HSD1 in macrophages could therefore be crucial to the ability to shape an ongoing inflammatory response, either through intracellular regeneration of glucocorticoids or indirectly by diversion of glucose-6-phosphate (Fig. 2). Evidence for dynamic regulation of 11β-HSD1 during an inflammatory response *in vivo* comes from the rapid induction of 11β-HSD1 activity in neutrophils and monocytes/macrophages during sterile peritonitis in mice; 11β-HSD1 activity decreases as the inflammation resolves [1], [36]. The latter is possibly an active process; 11β-HSD1 activity is rapidly down-regulated in macrophages that have phagocytosed apoptotic neutrophils [35], a highly pro-resolution process [50]. This reasoning led to the hypothesis that the early induction of 11β-HSD1 in macrophages increases glucocorticoid action within these cells, promoting an anti-inflammatory phenotype and leading to more rapid resolution of inflammation [1], [51].

### 1.4 11β-HSD1 in acute inflammation – regulation

In most animal models of acute inflammation 11β-HSD1 activity is up-regulated in the inflamed tissue, whereas 11β-HSD2 (if expressed at all) is down-regulated. This is true of the inflamed colon and the arthritic joint [52], [53], [54], but not the vasculature [55]. This switch in the balance of 11β-HSD1 and 2 activities is predicted to increase paracrine/autocrine glucocorticoid action, though this has not been directly tested. Induction of 11β-HSD1 (and repression of 11β-HSD2) at inflamed sites is probably due to local release of the pro-inflammatory cytokines IL-1 and TNFα which stimulate transcription of the 11β-HSD1 gene promoter through increased binding of the transcriptional regulators CCAAT/enhancer binding protein (C/EBP)-β and nuclear factor kappa-light-chain-enhancer of activated B cells (NF-κB) [56], [57], [58] and repress the 11β-HSD2 gene promoter through an early growth response (EGR)-1 and NF-κB-dependent mechanism [59]. Normally glucocorticoids antagonise TNF-α or IL-1 action, but they act together with the pro-inflammatory cytokines to synergistically increase 11β-HSD1 expression in a variety of cell types [60], [61], [62], [63]. This is predicted to amplify the effect of glucocorticoid within a given cell or tissue, more rapidly promoting the repair and resolution phase. Whether 11β-HSD1 expression in inflammatory cells is regulated by similar mechanisms is an interesting question. Neither TNF-α nor IL-1β affect 11β-HSD1 activity in monocytes [37] and the signalling pathways that regulate macrophage 11β-HSD1 expression have not been characterised. C/EBPβ, a key regulator of 11β-HSD1 transcription in a variety of cell types [56], [57], [64], [65], [66], [67], [68], mediates M2 polarisation and arginase expression [69] yet also plays a role in pro-inflammatory cytokine expression in M1 macrophages [70]. However, genetic deletion of C/EBPβ abolishes both the liver-enriched inhibitor protein (LIP) and liver-enriched activator protein (LAP) C/EBPβ isoforms, the balance of which potently influences 11β-HSD1 mRNA levels *in vivo* [71] and also regulates osteoclast differentiation [72], a process akin to macrophage differentiation. The C/EBPβ-LIP:LAP ratio is regulated by mTOR [73], an integrator of cellular nutrient and energy metabolism, that is downstream of phosphatidylinositol 3-kinase (PI3K) and Akt, both capable of polarising macrophages [74], [75]. Plausibly, the C/EBPβ-LIP:LAP ratio differs according to the activating stimulus and may govern the expression level of 11β-HSD1 in polarised macrophages. The coupling within the endoplasmic reticulum of 11β-HSD1 activity to the supply of NADP(H) cofactor generated by hexose-6-phosphate dehydrogenase (H6PD) [76], [77], [78] is particularly intriguing in this respect, as it raises the possibility that cellular glucose availability and flux through the endoplasmic reticulum pentose phosphate pathway (the first 2 steps of which are catalysed by H6PD) controls 11β-HSD1 activity [79] which may therefore differ irrespective of expression levels in M1 and M2 macrophages.

### 1.5 11β-HSD1 in acute inflammation – function

**Fig. 3:**
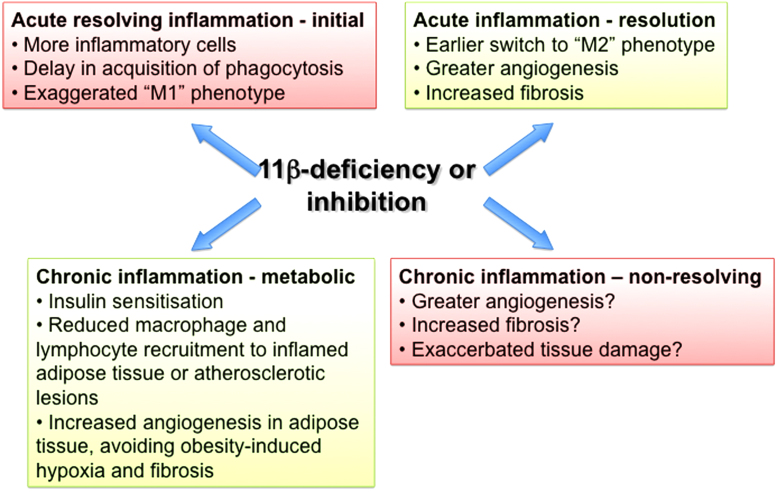
Effects of 11β-HSD1 deficiency/inhibition on acute and chronic inflammation. Deficiency or inhibition of 11β-HSD1 worsens or exaccerbates acute inflammation, but may also promote its successful resolution. During chronic metabolic inflammation (obesity, atherosclerosis, diabetes), 11β-HSD1 deficiency/inhibition is beneficial, reducing inflammatory cell recruitment to sites of inflammation and promoting insulin sensitisation. However, during chronic non-resolving inflammation, the pro-angiogenic, pro-fibrotic phenotype of 11β-HSD1 deficiency/inhibition may worsen tissue damage (see text for details).

Based on the expression of 11β-HSD1 in macrophages, its induction early during an inflammatory response and the well-known anti-inflammatory effects of glucocorticoids, it was anticipated that 11β-HSD1 deficiency or inhibition would attenuate local glucocorticoid production and thus worsen acute inflammation. This is indeed what is seen in 11β-HSD1-deficient (*Hsd11b1*^−/−^) mice, with more severe LPS-induced endotoxaemia (classically repressed by glucocorticoids [3], [80]), an earlier onset of inflammation in the K/BxN serum transfer model of inflammatory arthritis and more inflammatory cells (both neutrophils and monocytes/macrophages) recruited in sterile peritonitis or pleuritis and in the injured myocardium following myocardial infarction [81], [82], [83] (and see Fig. 3). This increase in inflammation could reflect greater recruitment and/or delayed clearance/apoptosis of neutrophils [1], [84]. In support of the latter, *Hsd11b1*^*−/−*^ mice show delayed macrophage acquisition of phagocytic capacity for apoptotic neutrophils as well as an increase in the number of free apoptotic neutrophils during sterile peritonitis, although surprisingly the peritonitis resolves at the same time as in wild-type mice [1]. Also surprising was the finding that despite the increased inflammation early following myocardial infarction or possibly because of it, heart function post-infarction is much better preserved in *Hsd11b1*^*−/−*^ mice than in controls. Underlying the improved recovery from myocardial infarction is an increased angiogenic response to injury [85], probably as a consequence of an earlier accumulation of reparative M2 (Ym1^+^) macrophages and higher levels of the pro-angiogenic cytokine IL-8 in the hearts of *Hsd11b1*^*−/−*^ mice [82]. It will be important to determine how generally this accelerated switch in macrophage phenotype from M1 to M2 applies to inflammation in *Hsd11b1*^*−/−*^ mice; so far it has only been reported in myocardial infarction and M2-like polarisation is not a general feature of 11β-HSD1-deficient macrophages, at least *in vitro* [1], [81] or *in vivo*, in adipose tissue of high fat fed obese mice [86]. Despite the lack of detectable difference in adipose tissue macrophage phenotype, an increased angiogenic response to tissue ischaemia is also seen in adipose tissue of obese *Hsd11b1*^*−/−*^ mice and underlies their resistance to some of the adverse metabolic consequences of obesity [87], suggesting the pro-angiogenic phenotype may be at least partly independent of macrophages.

How is the improved recovery of *Hsd11b1*^*−/−*^ mice from inflammation following myocardial infarction reconciled with our original hypothesis? As predicted by the hypothesis, deficiency in 11β-HSD1 causes greater release of pro-inflammatory cytokines from LPS-treated macrophages [1], [81], suggesting an exaggerated M1 macrophage phenotype. However, the earlier switch to an M2 phenotype was unexpected. Whether this reflects a switch to M2 phenotype *in situ* or recruitment of a distinct subset of monocytes is currently unknown. It is possible that this is a consequence of prolonged activation of the HPA axis in *Hsd11b1*^*−/−*^ mice. However, these mice show little perturbation of plasma corticosterone levels, even following stress, on this genetic background [88], so the earlier switch is unlikely to be mediated by plasma glucocorticoids. Moreover, as discussed above, intracellular amplification of glucocorticoid signalling by 11β-HSD1 is predicted to accelerate repair and resolution processes, not attenuate them. Several key factors implicated in macrophage polarisation [89] are differentially expressed in *Hsd11b1*^*−/−*^ mice. The Src homology 2-containing inositol-5′-phosphatase (SHIP)-1 negatively regulates the PI3K pathway. It represses the generation of M2 macrophages [74] yet restrains LPS-induced (M1) activation of bone marrow-derived (naïve) macrophages [90]. Moreover, elevated SHIP1 expression induces endotoxin tolerance [90] with reduced pro-inflammatory cytokine production with subsequent endotoxin challenge [90]. The increased LPS-responsiveness of thioglycollate elicited peritoneal (TEP) macrophages from *Hsd11b1*^*−/−*^ mice was attributed to elevated SHIP1 levels as a consequence of higher levels of TGFβ [81] though SHIP1 levels appear to decrease more rapidly following LPS in *Hsd11b1*^*−/−*^ macrophages than in wild-type. In spleenic macrophages, basal SHIP1 levels are normal in *Hsd11b1*^*−/−*^ mice, but unlike wild-type spleenic macrophages, those from *Hsd11b1*^*−/−*^ mice fail to down-regulate SHIP1 following LPS [81]. Whether this induces endotoxin tolerance [90] to a greater extent in *Hsd11b1*^*−/−*^ macrophages is something that requires testing. Thus, SHIP1 appears abnormally regulated in *Hsd11b1*^*−/−*^ macrophages, though why is currently unclear. Nevertheless, these somewhat confusing data illustrate that M1/M2 macrophage polarisation in *Hsd11b1*^*−/−*^ mice may be highly dependent upon the macrophage population and context.

Hypoxia-inducible factor (HIF1)-α, which promotes M1 polarisation, is decreased in adipose tissue of *Hsd11b1*^*−/−*^ mice, whereas levels of PPARγ (which promotes the M2 phenotype) are increased [87], [91]. Whether these factors are differentially expressed in macrophages of *Hsd11b1*^*−/−*^ mice will be important to determine.

The outcome of acute inflammation is not invariably improved in *Hsd11b1*^*−/−*^ mice. At the stage when arthritis has largely resolved in wild-type mice following K/BxN serum transfer, joints of *Hsd11b1*^*−/−*^ mice show greater periarticular fibrosis, more extensive exostoses and ganglion cyst formation. Following carageenan-induced pleurisy, *Hsd11b1*^*−/−*^ mice show persistence of inflammation at a stage when it is resolving in wild-type controls, as well as lymphoid aggregates within the lung and formation of fibrous adhesions between lung lobes, the latter not present in control mice [83]. Whether these disadvantageous features result from greater inflammation in *Hsd11b1*^*−/−*^ mice, an earlier switch to a pro-repair (pro-fibrotic) M2 phenotype, a greater response of the non-immune tissue or a combination of all of these will be an interesting question for the future. Moreover, the consequences of more extended inflammation will be interesting to determine. The preliminary findings in arthritis and carageenan induced pleurisy suggest that 11β-HSD1-deficiency or inhibition may aggravate diseases associated with a dysregulated angiogenic and pro-fibrotic phenotype, including rheumatoid arthritis.

### 1.6 Chronic inflammation

Chronic inflammation results from a failure to resolve acute inflammation. Atherosclerosis, diabetes, metabolic syndrome and Alzheimer's disease are all now recognised as chronic inflammatory diseases. Even simple obesity is frequently associated with low level chronic inflammation within the adipose tissue. The elevation in systemic pro-inflammatory cytokines during chronic inflammation might be expected to activate the HPA axis. However, plasma cortisol is normal in both the “classic” inflammatory diseases (rheumatoid arthritis, inflammatory bowel disease, *etc.*) and in the “metabolic” inflammatory diseases (atherosclerosis, metabolic syndrome, diabetes), at least until these become complicated by additional pathologies. HPA axis activity may be elevated in metabolic inflammation, with increased clearance of glucocorticoids maintaining normal plasma cortisol levels [92] but possibly increasing plasma cortisone levels (and thus 11β-HSD1 substrate), though this has only been indirectly measured. In rheumatoid arthritis and other inflammatory diseases however, the HPA axis appears relatively suppressed, especially given the level of systemic inflammation expected to activate the axis [4], [93]. Edwards has recently hypothesised that this apparent deficiency in HPA axis activation is a result of the systemic increase in TNF-α in chronic inflammation inducing a widespread increase in 11β-HSD1 expression, including in the hypothalamus, thus amplifying negative feedback by glucocorticoids on the HPA axis [93]. Whether this is indeed the case requires experimental testing, but consistent with this hypothesis, whole body conversion of cortisone to cortisol (relative to cortisol to cortisone) is increased in patients with inflammatory disease [94] suggesting altered balance of 11β-HSD activities in favour of 11β-reductase (11β-HSD1).

### 1.7 Metabolic syndrome, type 2 diabetes and atherosclerosis

11β-HSD1 deficiency or inhibition is metabolically beneficial in rodent models of diet-induced obesity or diabetes. It improves hepatic and adipose insulin sensitivity, attenuates hepatic gluconeogenesis, skews to a “cardioprotective” plasma lipid profile, shifts hepatic lipid metabolism from lipogenesis to fatty acid oxidation and causes a preferential gain of peripheral adipose tissue at the expense of visceral [86], [91], [95], [96], [97], [98], [99], [100], [101] (and see Fig. 3). Similarly, in patients with type 2 diabetes, 11β-HSD1 inhibition lowers plasma glucose and lipids, consistent with rodent studies. It also modestly reduces blood pressure in human hypertension [102], [103], [104]. Intriguingly, an 11β-HSD1 inhibitor more effectively improved glucose homeostasis in obese mice when administered close to the time of the diurnal peak of plasma glucocorticoid levels [105]. Given that 11β-HSD1 mRNA probably does not vary with the circadian rhythm [105], [106] (though one study suggests it may in rats [107]), this is much more likely to reflect high 11β-HSD1 substrate levels at peak HPA axis activity [108]. Indeed, 11β-HSD1 may contribute to normal circadian control of the HPA axis, at least in some genetic backgrounds [88], [108]. 11β-HSD1 is expressed in the paraventricular nucleus of the human hypothalamus, suggesting a conserved role in HPA axis regulation [109].

Recent data suggest that the liver is not the sole or even predominant target of the metabolically beneficial effects of 11β-HSD1-deficiency or inhibition; conditional deletion of 11β-HSD1 in hepatocytes of mice produces only minimal improvements in glucose homeostasis in diet-induced obesity [110]. Instead, increased glucocorticoid activity in adipose tissue is implicated. In obese humans, numerous studies have reported elevated 11β-HSD1 expression in subcutaneous adipose tissue (reviewed in Ref. [92]) and in human omental fat, 11β-HSD1 expression correlates with adipocyte hypertrophy [111], [112], itself associated with a more pro-inflammatory state [113], [114]. In mice, a two to three-fold elevation of 11β-HSD1 selectively in adipose tissue phenocopies the metabolic syndrome, with central obesity, insulin resistance, dyslipidaemia and hypertension [115], [116] whereas similar transgenic expression of 11β-HSD2 in adipocytes (it is not normally expressed in adipocytes), presumably lowering intra-adipose glucocorticoid action, causes insulin sensitisation in high fat fed mice [117].

11β-HSD1-deficiency protects against pro-inflammatory changes in adipose tissue in obesity. Inflammatory cell (macrophages, lymphocytes) infiltration of mesenteric adipose tissue is lower in high fat-fed 11β-HSD1-deficient mice than in controls, probably due to reduced adipocyte secretion of the pro-inflammatory chemokine, monocyte chemoattractant-1 (MCP-1) [86]. This is associated with higher levels of AMP-activated protein kinase activation in this depot [86], likely to contribute to the maintained lipid oxidation with obesity [118] in 11β-HSD1-deficiency. Whether these changes are a cause or a consequence of the increase in angiogenesis and reduction in hypoxia and fibrosis recently described in the adipose tissue of these mice [87] is an interesting question. Adipose tissue hypoxia is associated with a local pro-inflammatory environment and leads to fibrosis though not necessarily angiogenesis [119], [120], [121], suggesting that it is the greater angiogenic response in *Hsd11b1*^*−/−*^ mice that is protective against adipose tissue hypoxia and fibrosis. PPARγ mRNA levels are higher and the pro-angiogenic response to PPARγ activation is much greater in *Hsd11b1*^*−/−*^ adipocytes than in controls, placing the adipocyte at the heart of the response. Whether there are also beneficial roles for macrophage and/or vascular 11β-HSD1 is important to determine.

As well as improving metabolic risk factors, deficiency in or inhibition of 11β-HSD1 also reduces atherosclerosis and systemic inflammation and lowers macrophage and T cell infiltration of atherosclerotic lesions in *Apoe*^*−/−*^ mice [122], [123], [124]. This is the converse of what happens with 11β-HSD2-deficiency, which is pro-inflammatory in the endothelium and accelerates atherosclerosis in *Apoe*^*−/−*^ mice, an effect at least partly mediated through activation of the MR as it is blocked by eplerenone, an MR antagonist [125]. The atheroprotective effects of 11β-HSD1-deficiency are likely to be mediated through both systemic (reduced circulating monocyte chemotactic protein (MCP)-1 and number of pro-inflammatory Ly6C^hi^ monocytes) and local (reduced aortic vascular cell adhesion molecule (VCAM)-1 expression) mechanisms [124]. It is interesting to speculate that reduced visceral adipose tissue inflammation may contribute to the reduction in systemic inflammation – as in diet-induced obesity, mesenteric adipose tissue MCP-1 mRNA levels are reduced in western diet-fed 11β-HSD1-deficient *Apoe*^*−/−*^ mice [124].

### 1.8 “Classic” inflammatory diseases – rheumatoid arthritis

If 11β-HSD1-deficiency is beneficial in chronic “cardiometabolic inflammation”, what of the classical inflammatory diseases, in which a glucocorticoid-insufficient state is suggested and glucocorticoid therapy remains highly effective? Inevitably, studies in animals are predominantly short term, modelling the disease, whereas the disease in patients frequently reflects years of accumulated damage and inflammation. These situations may be quite different. Nevertheless, accumulating evidence in both patients and animal models is consistent with dysregulated 11β-HSD1 in the inflamed joint in rheumatoid arthritis as well as increased colonic expression of 11β-HSD1 at sites of inflammation in inflammatory bowel disease (reviewed in Ref. [35]). So far, studies in inflammatory bowel disease have gone little beyond observation, though they do suggest that at least some of the increase in 11β-HSD1 expression occurs in activated lymphocytes that migrate from the inflamed colon to the draining lymph nodes [54]. Studies in human patients with rheumatoid arthritis suggest differential regulation of 11β-HSDs in immune and mesenchymal cells. Comparison of cortisone and cortisol levels in synovial fluid and serum suggest the balance favours intra-articular generation of cortisol in the rheumatic joint [126] although it seems that even so, the overall capacity to convert cortisone to cortisol may be reduced in the inflamed arthritic synovium compared to non-inflamed. However, within inflamed rheumatic joints, synovial inflammation still correlates with conversion of cortisone to cortisol [127]. This complex relationship probably reflects the balance between high expression of 11β-HSD1 in synovial fibroblasts from arthritic patients (almost certainly as a result of the pro-inflammatory cytokine environment) and expression of 11β-HSD2 in synovial macrophages from patients with rheumatoid arthritis [126], [127], [128]. This latter finding accords with other studies identifying 11β-HSD2 as a peripheral blood mononuclear cell marker of early rheumatoid arthritis and highly expressed in the arthritic joint [129], [130]. 11β-HSD2-positive macrophages have also been described in the lungs of patients who died of acute respiratory distress syndrome [131]. Similar cells (macrophages, lymphocytes) from healthy humans do not express 11β-HSD2 [37], [130], nor has 11β-HSD2 been found in mouse leukocytes [1]. 11β-HSD2 expression in leukocytes may reflect a species difference between mouse and human, or could, in humans, reflect an adaptive response to chronic inflammation. The biological reason for this apparently pro-inflammatory change is unknown but it is likely to cause resistance to endogenous glucocorticoids, which might be overcome by pharmacological levels of synthetic glucocorticoids like prednisolone or bypassed with non-metabolised synthetic glucocorticoids like dexamethasone.

What might 11β-HSD1 inhibition do in chronic inflammatory disease? If the Edwards hypothesis [93] is correct, then systemic inhibition of 11β-HSD1, particularly if administered during the night (in humans), should correct the HPA axis abnormality and boost the plasma cortisol levels. This might be enough to dampen down some of the inflammation, though 11β-HSD1 inhibition would also deprive inflamed tissues of the 11β-HSD1-mediated increase in intracellular glucocorticoid levels. Moreover, given that cortisol also activates MR (in the absence of 11β-HSD2), this could further exacerbate inflammation which could be particularly damaging within the vasculature (see below). In chronic inflammatory disease, continuing tissue injury is frequently associated with fibrosis and angiogenesis. Both may be exacerbated by 11β-HSD1 inhibition. As mentioned above, 11β-HSD1-deficient mice show an increased angiogenic response to adipose tissue hypoxia, to ischaemia following myocardial infarction, in wound healing and in sub-cutaneously implanted sponges [85], [87]. They also show a pro-fibrotic response to pleural inflammation and following inflammatory arthritis [83]. Whilst it is currently unclear whether the increased fibrosis in 11β-HSD1-deficient mice will resolve completely during recovery from inflammation, it is likely that if the injurious stimulus persists, fibrosis will be more severe with 11β-HSD1-deficiency or inhibition. In continuing liver injury, a population of macrophages with “M2”-like properties drives the fibrotic response, probably mediated at least in part through TGFβ1 [40]. Higher macrophage expression of TGFβ1 with 11β-HSD1-deficiency [81] may be an important contributor to the pro-fibrotic phenotype of these mice.

### 1.9 Glucocorticoid receptor or mineralocorticoid receptor activation?

Activation of MR, most notably in the heart and vasculature, has pro-inflammatory and pro-fibrotic consequences [132], [133]. Unlike synthetic glucocorticoids, most of which show selectivity for GR over MR, endogenous glucocorticoids bind with higher affinity to MR than to GR. Thus, MR is usually considered near saturated at circulating glucocorticoid levels, even at the diurnal nadir [134]. Aldosterone activates MR irrespective of which cells it is expressed in, but cortisol activation of MR is normally prevented if 11β-HSD2 is co-expressed with MR. However, under conditions of oxidative stress, endogenous glucocorticoids can activate MR, at least in the cardiovascular system [135]. A crucial question therefore, central to the function of 11β-HSD1, is which receptor binds the ligand it generates, GR or MR? This may differ according to tissues. MR is absent from liver, so in this tissue, 11β-HSD1 provides ligand to GR. However, MR is expressed in some classical glucocorticoid targets, including adipocytes and macrophages, normally in the absence of 11β-HSD2, where it presumably functions as a glucocorticoid receptor. A pro-inflammatory role for glucocorticoid-activated MR is suggested; eplerenone treatment of *ob/ob* mice prevented the obesity-associated increases in MCP-1, TNF-α and other inflammatory markers in adipose tissue [136]. Whether the relevant cell is the adipocyte, however, is unclear. Whereas MR activation (presumably by glucocorticoids) in macrophages appears pro-inflammatory, macrophage-specific deletion of MR appears anti-inflammatory – it causes M2 polarisation of macrophages [43] and reduces cerebral infarct area following ischaemia in mice, concomitant with reduced expression of M1 macrophage markers (TNF-α, IL-1, MCP-1, *etc.*) but maintained M2 markers (Ym1, Arg1) [137]. Thus, the consequences of 11β-HSD1-mediated glucocorticoid generation could differ greatly, depending on cellular oxidation/stress state and the relative levels of GR *versus* MR.

## 2 Summary and conclusions

Consistent with the adverse metabolic effects of glucocorticoid excess, 11β-HSD1 deficiency or inhibition is clearly beneficial in cardiometabolic disease. The extent to which this is dependent on inhibition/deficiency within inflammatory cells will be interesting to discover. Also, whether 11β-HSD1 deficiency/inhibition is beneficial in other types of inflammation remains to be seen. Current evidence suggests that the acute response to injury is more severe. The subsequent recovery phase may depend on whether the injurious stimulus persists as in patients with rheumatoid arthritis (in which case 11β-HSD1 deficiency/inhibition may worsen the disease), or whether recovery and tissue remodelling occur, as for example follows myocardial infarction (when 11β-HSD1 deficiency/inhibition may aid recovery). The application of Cre/Lox technology to generate tissue- and cell-specific “knock-out” of 11β-HSD1 will be invaluable in dissecting the contributions of immune cells, particularly macrophages and neutrophils, to the pro-angiogenic and pro-fibrotic phenotype. In the future, such studies could lead to better targeting of glucocorticoid therapy, perhaps even targeting macrophages separately from host tissues at specific temporal stages of disease. As already suggested [1], targeted delivery of inactive glucocorticoid precursors to macrophages might provide an effective future therapy for chronic inflammatory disease.

## Disclosures

Jonathan R. Seckl holds IP on the use of 11β-HSD1 inhibitors in diabetes, atherosclerotic disease and age-associated cognitive impairment. None of the other authors have anything to disclose.
